# Supplementary material for: Conditions for transient epidemics of waterborne disease in spatially explicit systems
Source: R Soc Open Sci. 2019 May 22;6(5):181517. doi: 10.1098/rsos.181517 (PMC6549988; doi:10.1098/rsos.181517)
Supplement: Supplementary appendices and figures [file rsos181517supp1.pdf]

Conditions for transient epidemics of waterborne disease in spatially explicit systems

Supplementary Material

Lorenzo Mari[1,†], Renato Casagrandi[1], Enrico Bertuzzo[2],
Andrea Rinaldo[3,4], Marino Gatto[1]

[1] Dipartimento di Elettronica, Informazione e Bioingegneria,
Politecnico di Milano, 20133 Milano, Italy

[2] Dipartimento di Scienze Ambientali, Informatica e Statistica,
Università Ca' Foscari Venezia, 30170 Venezia Mestre, Italy

[3] Laboratory of Ecohydrology,
Ecole Polytechnique Fédérale de Lausanne, 1015 Lausanne, Switzerland

[4] Dipartimento ICEA,
Università di Padova, 35131 Padova, Italy

[†] Corresponding author: lorenzo.mari@polimi.it

Contents

A Conditions for long-term pathogen invasion and establishment **2**

B Derivation of conditions for transient epidemicity **4**

C Numerical evaluation of endemicity and epidemicity conditions **7**

D Supplementary figures **13**

A Conditions for long-term pathogen invasion and establishment

Conditions for waterborne pathogen establishment in spatially extended systems (river networks, in particular) have already been provided elsewhere (Gatto et al., 2012, 2013; see also Eisenberg et al., 2013; Tien et al., 2015). Here we recall some of the main results that are useful to complement the analysis of the conditions for short-term pathogen outbreak.

Pathogens can invade the system and establish therein in the long run if and only if the disease-free equilibrium (DFE) of system (1) in the main text is asymptotically unstable, i.e. if the dominant eigenvalue of the Jacobian matrix $\mathbf{J_0}$ (the $3n \times 3n$ state matrix describing the dynamics of the system close to the DFE equilibrium) is positive. It is straightforward to verify that

$$\mathbf{J_0} = \begin{bmatrix} -\mu\mathbf{U_n} & \mathbf{0_n} & -\left[\left(\mathbf{U_n} - \mathbf{m^S}\right)\mathbf{N} + \mathbf{m^S}\mathbf{N}\mathbf{Q}\right]\boldsymbol{\beta} \\ \mathbf{0_n} & -\phi\mathbf{U_n} & \left[\left(\mathbf{U_n} - \mathbf{m^S}\right)\mathbf{N} + \mathbf{m^S}\mathbf{N}\mathbf{Q}\right]\boldsymbol{\beta} \\ \mathbf{0_n} & \boldsymbol{\theta}\mathbf{W}^{-1}\left(\mathbf{U_n} - \mathbf{m^I} + \mathbf{Q}^T\mathbf{m^I}\right) & -\boldsymbol{\nu} - \left(\mathbf{U_n} - \mathbf{W}^{-1}\mathbf{P}^T\mathbf{W}\right)\mathbf{l} \end{bmatrix},$$

where: $\mathbf{U_n}$ is the identity matrix of dimension n; $\phi = \mu + \delta + \gamma$; and $\mathbf{N}$, $\mathbf{W}$, $\boldsymbol{\beta}$, $\boldsymbol{\theta}$, $\boldsymbol{\nu}$, $\mathbf{m^S}$, $\mathbf{m^I}$, $\mathbf{l}$ are diagonal matrices with positive entries corresponding to the parameters N_i, W_i, β_i, $\theta_i = p_i/K$, ν_i, m_i^S, m_i^I, l_i, with $i = 1, \cdots, n$. Indeed, by noting the block-triangular structure of $\mathbf{J_0}$, one can immediately observe that the Jacobian has n eigenvalues equal to $-\mu$. Therefore, the stability properties of the DFE can be determined based on the eigenvalues of the submatrix

$$\mathbf{J_0'} = \begin{bmatrix} -\phi\mathbf{U_n} & \left[\left(\mathbf{U_n} - \mathbf{m^S}\right)\mathbf{N} + \mathbf{m^S}\mathbf{N}\mathbf{Q}\right]\boldsymbol{\beta} \\ \boldsymbol{\theta}\mathbf{W}^{-1}\left(\mathbf{U_n} - \mathbf{m^I} + \mathbf{Q}^T\mathbf{m^I}\right) & -\boldsymbol{\nu} - \left(\mathbf{U_n} - \mathbf{W}^{-1}\mathbf{P}^T\mathbf{W}\right)\mathbf{l} \end{bmatrix}.$$

$\mathbf{J_0'}$ is a Metzler matrix and its associated graph is strongly connected (see main text), thus its dominant eigenvalue $\lambda_{\max}^J$ is simple and real (Horn and Johnson, 1990). A transcritical bifurcation of the DFE occurs when $\lambda_{\max}^J = 0$, namely when the determinant of $\mathbf{J_0'}$ is zero (Kuznetsov, 1995). As long as the DFE is stable, all the eigenvalues have negative real parts and the determinant of $\mathbf{J_0'}$ is positive because $\mathbf{J_0'}$ is a matrix of order $2n$. The DFE thus becomes unstable when the determinant of $\mathbf{J_0'}$

switches from positive to negative. Applying Silvester's (2000) theorem for the determinant of block matrices, we can evaluate the determinant of $\mathbf{J_0'}$ as

$$\det(\mathbf{J_0'}) = \det\left(\phi\boldsymbol{\nu} + \phi\left(\mathbf{U_n} - \mathbf{W}^{-1}\mathbf{P}^T\mathbf{W}\right)\mathbf{l} + \right. \\ \left. - \boldsymbol{\theta}\mathbf{W}^{-1}\left(\mathbf{U_n} - \mathbf{m^I} + \mathbf{Q}^T\mathbf{m^I}\right)\left[\left(\mathbf{U_n} - \mathbf{m^S}\right)\mathbf{N} + \mathbf{m^S}\mathbf{N}\mathbf{Q}\right]\boldsymbol{\beta}\right) .$$

By introducing the matrices (Gatto et al., 2012, 2013)

$$\begin{aligned}
\mathbf{T_0'} &= \boldsymbol{\nu}^{-1}\left(\mathbf{W}^{-1}\mathbf{P}^T\mathbf{W} - \mathbf{U_n}\right)\mathbf{l}, \\
\mathbf{R_0} &= \frac{1}{\phi}\boldsymbol{\nu}^{-1}\boldsymbol{\theta}\mathbf{W}^{-1}\left(\mathbf{U_n} - \mathbf{m^I}\right)\left(\mathbf{U_n} - \mathbf{m^S}\right)\mathbf{N}\boldsymbol{\beta}, \\
\mathbf{R_0^S} &= \frac{1}{\phi}\boldsymbol{\nu}^{-1}\boldsymbol{\theta}\mathbf{W}^{-1}\left(\mathbf{U_n} - \mathbf{m^I}\right)\mathbf{m^S}\mathbf{N}\mathbf{Q}\boldsymbol{\beta}, \\
\mathbf{R_0^I} &= \frac{1}{\phi}\boldsymbol{\nu}^{-1}\boldsymbol{\theta}\mathbf{W}^{-1}\mathbf{Q}^T\mathbf{m^I}\left(\mathbf{U_n} - \mathbf{m^S}\right)\mathbf{N}\boldsymbol{\beta}, \\
\mathbf{R_0^{SI}} &= \frac{1}{\phi}\boldsymbol{\nu}^{-1}\boldsymbol{\theta}\mathbf{W}^{-1}\mathbf{Q}^T\mathbf{m^I}\mathbf{m^S}\mathbf{N}\mathbf{Q}\boldsymbol{\beta} \quad \text{and} \\
\mathbf{R_0^\star} &= \mathbf{R_0} + \mathbf{R_0^S} + \mathbf{R_0^I} + \mathbf{R_0^{SI}},
\end{aligned}$$

the instability condition $\det(\mathbf{J_0'}) < 0$ can also be written as $\det\left(\mathbf{U_n} - \mathbf{T_0'} - \mathbf{R_0^\star}\right) < 0$, which is equivalent to requiring that the dominant eigenvalue $\mathcal{R}_0$ of matrix $\mathbf{G_0} = \mathbf{T_0'} + \mathbf{R_0^\star}$ is larger than one. Actually, the DFE becomes unstable, thus allowing for the onset of endemic pathogen transmission, if $\mathcal{R}_0$ switches from being less than one to being larger than one. In the absence of hydrologic transport and human mobility we have $\mathbf{G_0} = \mathbf{R_0}$, and the instability condition of the DFE reduces to

$$\max_i \frac{\beta_i\theta_i N_i}{\phi\nu_i W_i} > 1 ,$$

which corresponds to the instability condition for a system made of n isolated communities (i.e. local basic reproduction number larger than one; see e.g. Codeço, 2001; Gatto et al., 2013).

We finally recall that the analysis of the dominant eigenvector of matrix $\mathbf{J_0'}$ (or $\mathbf{G_0}$) also provides important indications about the unfolding of the outbreak (Gatto et al. 2012, 2013; see also Mari

et al. 2014 for an extension to periodically forced systems). In particular, the dominant eigenvector of $\mathbf{J_0'}$ (which has strictly positive components) pinpoints the direction in the state space along which the system orbit, after a transient due to the initial perturbation, will converge to (asymptotically stable DFE) or diverge from (asymptotically unstable DFE) the equilibrium. The components of the dominant eigenvector of $\mathbf{J_0'}$ correspond to the values of the infected individuals' abundances and the bacterial concentrations in the different human communities. This result obviously holds true only in a neighborhood of the DFE, where the dynamics of the system can be well represented through its linearization. If this is the case, an epidemic outbreak will thus mainly propagate (or fade away, in case of a stable DFE) along the locations that correspond to the largest components of the dominant eigenvector of matrix $\mathbf{J_0'}$ as soon as the effects of the initial perturbation vanish.

B Derivation of conditions for transient epidemicity

Mari et al. (2017) showed that postulating the existence of perturbations for which condition (2) in the main text is verified amounts to requiring that

$$\lambda_{\max}\left(H(\mathbf{C}^T\mathbf{C}\mathbf{J_0})\right) > 0\,, \tag{S1}$$

where: $\lambda_{\max}(\cdot)$ indicates the dominant eigenvalue of a matrix; $H(\mathbf{A}) = (\mathbf{A} + \mathbf{A}^T)/2$ is the Hermitian part of a generic matrix $\mathbf{A}$; $\mathbf{C}$ is the $q \times 3n$ output transformation matrix defined in the main text (equation (3)); and $\mathbf{J_0}$ is the Jacobian matrix of system (1) (main text) at the DFE (see Appendix A). In general, inequality (S1) provides a simple test to detect g-reactivity; in this specific application, it represents the (necessary, yet not sufficient) condition for the occurrence of a transient epidemic wave. In fact, not all generic perturbations to a stable, g-reactive DFE will initially be amplified in the system output. Perturbations $\mathbf{x_0}$ for which condition (2) (main text) is verified define the so-called g-reactivity (or transient epidemicity, in this case) basin of the DFE (Mari et al., 2017, 2018).

It is also important to note that for model (1) in the main text $\ker(\mathbf{C}) = \ker(\mathbf{C}\mathbf{J_0})$, i.e. that the kernel (or null space) of the output matrix, defined as the set $\mathbf{z}$ of solutions to $\mathbf{C}\mathbf{z} = \mathbf{0}$, is the same as

the kernel of matrix $\mathbf{CJ_0}$. In this case, Mari et al. (2017) showed that the maximum initial growth rate of any generic perturbations to the DFE (indeed, what Neubert and Caswell, 1997, originally defined as reactivity in their isotropic framework) is given by $\lambda_{\max}\left(H(\mathbf{CJ_0C^+})\right)$, where $\mathbf{C}^+ = \mathbf{C}^T(\mathbf{CC}^T)^{-1}$ is the right pseudo-inverse of matrix $\mathbf{C}$, that is a generalization of a matrix inverse (in fact, $\mathbf{CC}^+ = \mathbf{U_q}$, with $\mathbf{U_q}$ being the identity matrix of size q; note that $\mathbf{CC}^T$ is invertible because $\mathbf{C}$ is full rank). Therefore, $\lambda_{\max}(\mathbf{H_0}) > 0$ (condition (4) in the main text), with

$$\mathbf{H_0} = H(\mathbf{CJ_0C^+}) = \frac{1}{2}(\mathbf{CJ_0C^+} + (\mathbf{C}^+)^T\mathbf{J_0}^T\mathbf{C}^T) = \begin{bmatrix} -\boldsymbol{\mathcal{F}} & \boldsymbol{\mathcal{H}} \\ \boldsymbol{\mathcal{H}} & -\boldsymbol{\mathcal{G}} \end{bmatrix}$$

where

$$\boldsymbol{\mathcal{F}} = \phi\mathbf{U_n}$$

$$\boldsymbol{\mathcal{G}} = \boldsymbol{\nu} + \mathbf{c_B}\left(\mathbf{U_n} - \mathbf{W}^{-1}\mathbf{P}^T\mathbf{W}\right)\mathbf{l}\,\mathbf{c_B}^{-1} \quad \text{and}$$

$$\boldsymbol{\mathcal{H}} = \frac{1}{2}\left\{\mathbf{c_I}\left[\left(\mathbf{U_n} - \mathbf{m^S}\right)\mathbf{N} + \mathbf{m^S}\mathbf{NQ}\right]\boldsymbol{\beta}\mathbf{c_B}^{-1} + \mathbf{c_B}\boldsymbol{\theta}\mathbf{W}^{-1}\left(\mathbf{U_n} - \mathbf{m^I} + \mathbf{Q}^T\mathbf{m^I}\right)\mathbf{c_I}^{-1}\right\},$$

represents an alternative test for transient epidemicity. Note that conditions (S1) and (4) in the main text are completely equivalent for the problem at hand; however, the latter might be preferred in practice because $H(\mathbf{C}^T\mathbf{CJ_0})$ has higher dimension ($3n \times 3n$) than $\mathbf{H_0}$ ($2n \times 2n$). Matrix $\mathbf{H_0}$ is Hermitian, thus its eigenvalues (and in particular $\lambda^H_{\max}$) are real (Horn and Johnson, 1990). Also, the off-diagonal entries of $\mathbf{H_0}$ are all nonnegative and at least one diagonal entry is negative, thus $\mathbf{H_0}$ is a proper Metzler matrix (Horn and Johnson, 1990). If we assume that the union of the graphs associated with $\mathbf{P}$ and $\mathbf{Q}$ is strongly connected (so that the graph associated with $\mathbf{H_0}$ is strongly connected too), we can apply the Perron-Frobenius theorem for irreducible matrices and conclude that $\lambda^H_{\max}$ is a simple real root of the characteristic polynomial (see again Horn and Johnson, 1990). As long as the DFE is non-g-reactive, all the eigenvalues of $\mathbf{H_0}$ have negative real parts and the determinant of $\mathbf{H_0}$ is positive because $\mathbf{H_0}$ is a matrix of order $2n$. Thus, the DFE becomes g-reactive when the determinant of $\mathbf{H_0}$ switches from positive to negative or, equivalently, when $\lambda^H_{\max}$ becomes larger than zero.

The g-reactivity properties of the DFE can actually be evaluated based on a matrix of reduced order n. We note in fact that $\boldsymbol{\mathcal{F}}$ is a positive scalar matrix, hence commuting with any other matrix – thus, in particular, with $\boldsymbol{\mathcal{H}}$. In this case, it can be shown (Silvester, 2000) that $\det(\mathbf{H_0}) = \det\left(\boldsymbol{\mathcal{F}}\boldsymbol{\mathcal{G}} - \boldsymbol{\mathcal{H}}^2\right)$. With lengthy, yet straightforward algebraic manipulations, the previous relation can be written as

$$\begin{aligned}
\det(\mathbf{H_0}) = \phi^n \det(\boldsymbol{\nu}) \det \Bigg(& \mathbf{U_n} - \boldsymbol{\nu}^{-1} \left(\mathbf{c_B}\mathbf{W}^{-1}\mathbf{P}^T\mathbf{W}\mathbf{c_B}^{-1} - \mathbf{U_n}\right) \mathbf{l} + \\
& - \frac{\boldsymbol{\nu}^{-1}}{4\phi} \left[\mathbf{c_I}\left(\mathbf{U_n} - \mathbf{m^S}\right)\mathbf{N}\boldsymbol{\beta}\mathbf{c_B}^{-1} + \mathbf{c_B}\boldsymbol{\theta}\mathbf{W}^{-1}\left(\mathbf{U_n} - \mathbf{m^I}\right)\mathbf{c_I}^{-1}\right]^2 + \\
& - \frac{\boldsymbol{\nu}^{-1}}{2\phi} \left[\mathbf{c_I}\left(\mathbf{U_n} - \mathbf{m^S}\right)\mathbf{N}\boldsymbol{\beta}\mathbf{c_B}^{-1} + \mathbf{c_B}\boldsymbol{\theta}\mathbf{W}^{-1}\left(\mathbf{U_n} - \mathbf{m^I}\right)\mathbf{c_I}^{-1}\right] \mathbf{c_I}\mathbf{m^S}\mathbf{N}\mathbf{Q}\boldsymbol{\beta}\mathbf{c_B}^{-1} + \\
& - \frac{\boldsymbol{\nu}^{-1}}{2\phi} \left[\mathbf{c_I}\left(\mathbf{U_n} - \mathbf{m^S}\right)\mathbf{N}\boldsymbol{\beta}\mathbf{c_B}^{-1} + \mathbf{c_B}\boldsymbol{\theta}\mathbf{W}^{-1}\left(\mathbf{U_n} - \mathbf{m^I}\right)\mathbf{c_I}^{-1}\right] \mathbf{c_B}\boldsymbol{\theta}\mathbf{W}^{-1}\mathbf{Q}^T\mathbf{m^I}\mathbf{c_I}^{-1} + \\
& - \frac{\boldsymbol{\nu}^{-1}}{4\phi} \left[\mathbf{c_I}\mathbf{m^S}\mathbf{N}\mathbf{Q}\boldsymbol{\beta}\mathbf{c_B}^{-1} + \mathbf{c_B}\boldsymbol{\theta}\mathbf{W}^{-1}\mathbf{Q}^T\mathbf{m^I}\mathbf{c_I}^{-1}\right]^2 \Bigg) .
\end{aligned}$$

By introducing the matrices $\mathbf{T_0}$, $\mathbf{E_0}$, $\mathbf{E_0^{SI}}$, $\mathbf{E_0^S}$ and $\mathbf{E_0^I}$ (see main text), the g-reactivity (or transient epidemicity) condition $\det(\mathbf{H_0}) < 0$ can be written as $\det\left(\mathbf{U_n} - \mathbf{T_0} - \mathbf{E_0^\star}\right) < 0$, with $\mathbf{E_0^\star} = \mathbf{E_0} + \mathbf{E_0^S} + \mathbf{E_0^I} + \mathbf{E_0^{SI}}$. Equivalently, the epidemicity condition is that the dominant eigenvalue $\mathcal{E}_0$ of matrix $\mathbf{F_0} = \mathbf{T_0} + \mathbf{E_0^\star}$ is larger than one. Actually, the DFE becomes g-reactive when $\mathcal{E}_0$ switches from being less than one to being larger than one. In this case, a transient epidemic outbreak can start, provided that the DFE is perturbed by a suitable injection of bacteria and/or infected human hosts, i.e. following a perturbation $[\mathbf{i}^T\ \mathbf{b}^T]^T$ lying in the g-reactivity basin of the equilibrium, which for the problem at hand is given by the quadratic inequality

$$\begin{bmatrix}\mathbf{i}^T & \mathbf{b}^T\end{bmatrix} \mathbf{H_0} \begin{bmatrix}\mathbf{i} \\ \mathbf{b}\end{bmatrix} = -\phi\mathbf{i}^T\mathbf{i} + 2\mathbf{b}^T\boldsymbol{\mathcal{H}}\mathbf{i} - \mathbf{b}^T\boldsymbol{\mathcal{G}}\mathbf{b} > 0 .$$

We also remark that in the absence of hydrologic transport and human mobility, i.e. if $\mathbf{l} = \mathbf{0_n}$ and $\mathbf{m^S} = \mathbf{m^I} = \mathbf{0_n}$ (so that $\mathbf{T_0} = \mathbf{0_n}$, $\mathbf{E_0^\star} = \mathbf{E_0}$, hence $\mathbf{F_0} = \mathbf{E_0}$), the epidemicity condition for the DFE

reduces to

$$\max_i \frac{\left(c_{I_i}{}^2 N_i \beta_i + c_{B_i}{}^2 \theta_i / W_i\right)^2}{4 c_{I_i}{}^2 c_{B_i}{}^2 \phi \nu_i} > 1\,,$$

as it can be found from g-reactivity analysis of n isolated communities (Mari et al., 2018).

As a corollary, we finally note that, close to the transient epidemicity boundary $\mathcal{E}_0 = 1$, the dominant eigenvector of matrix $\mathbf{F_0}$ corresponds to the spatial arrangement of the bacterial components of the dominant eigenvector of the Hermitian matrix $\mathbf{H_0}$, which in turn describes the geographical signature of the fastest growing perturbation to the DFE at time 0. In fact, the dominant eigenvector of $\mathbf{H_0}$ can be computed by solving

$$\mathbf{H_0} \begin{bmatrix} \mathbf{i} \\ \\ \mathbf{b} \end{bmatrix} = \lambda^H_{\max} \begin{bmatrix} \mathbf{i} \\ \\ \mathbf{b} \end{bmatrix}.$$

Recalling the block structure of $\mathbf{H_0}$, for $\lambda^H_{\max} \approx 0$ we get

$$-\boldsymbol{\mathcal{F}}\mathbf{i} + \boldsymbol{\mathcal{H}}\mathbf{b} = [0,\, 0,\, \cdots,\, 0]^T$$

$$\boldsymbol{\mathcal{H}}\mathbf{i} - \boldsymbol{\mathcal{G}}\mathbf{b} = [0,\, 0,\, \cdots,\, 0]^T.$$

From the first of the two equations above we have $\mathbf{i} = \boldsymbol{\mathcal{F}}^{-1}\boldsymbol{\mathcal{H}}\mathbf{b}$. Substituting this expression into the second we get $\boldsymbol{\mathcal{H}}\boldsymbol{\mathcal{F}}^{-1}\boldsymbol{\mathcal{H}}\mathbf{b} - \boldsymbol{\mathcal{G}}\mathbf{b} = [0,\, 0,\, \cdots,\, 0]^T$. Recalling that $\boldsymbol{\mathcal{F}}$ is a scalar (thus universally commuting) matrix, we have that $\left(\boldsymbol{\mathcal{F}}^{-1}\boldsymbol{\mathcal{H}}^2 - \boldsymbol{\mathcal{G}}\right)\mathbf{b} = -\boldsymbol{\mathcal{F}}^{-1}\left(\boldsymbol{\mathcal{F}}\boldsymbol{\mathcal{G}} - \boldsymbol{\mathcal{H}}^2\right)\mathbf{b} = [0,\, 0,\, \cdots,\, 0]^T$. From the algebraic derivation of epidemicity conditions we already know that $\boldsymbol{\mathcal{F}}\boldsymbol{\mathcal{G}} - \boldsymbol{\mathcal{H}}^2 = \boldsymbol{\mathcal{F}}\boldsymbol{\nu}(\mathbf{U_n} - \mathbf{F_0})$, which finally leads us to $\mathbf{F_0}\mathbf{b} = \mathbf{b}$. This corresponds to the equation for the evaluation of the dominant eigenvector of matrix $\mathbf{F_0}$ when its dominant eigenvalue $\mathcal{E}_0$ is (close to) one. We can thus conclude that at the epidemicity boundary the dominant eigenvector of $\mathbf{F_0}$ refers to the bacterial components of the state space. The components corresponding to infected hosts can be easily worked out as $\mathbf{i} = \boldsymbol{\mathcal{H}}\mathbf{b}/\phi$.

C Numerical evaluation of endemicity and epidemicity conditions

A simple MATLAB^{TM} implementation of the instructions needed to evaluate the asymptotic stability of the DFE of model (1) (main text) and its g-reactivity properties according to the output transfor-

mation defined in equation (3) (main text) is provided below. A network with four nodes, spatially homogeneous parameter values, and hydrologic transport and human mobility matrices

$$\mathbf{P} = \begin{bmatrix} 0 & 0 & 1 & 0 \\ 0 & 0 & 1 & 0 \\ 0.05 & 0.05 & 0 & 0.9 \\ 0 & 0 & 0.1 & 0 \end{bmatrix} \quad \text{and} \quad \mathbf{Q} = \begin{bmatrix} 0 & 0.5 & 0.4 & 0.1 \\ 0.3 & 0 & 0.5 & 0.2 \\ 1/3 & 1/3 & 0 & 1/3 \\ 0.2 & 0.2 & 0.6 & 0 \end{bmatrix}$$

is used as a proof-of-concept. In this example, the DFE is asymptotically stable ($\lambda_{\max}(\mathbf{J_0}) \approx -4.2 \cdot 10^{-5} < 0$), yet g-reactive ($\lambda_{\max}(\mathbf{H_0}) \approx 0.38 > 0$). A numerical simulation of the model (code also provided below) indeed shows that the prevalence of infected individuals may undergo transient growth before eventually fading out. More complex case studies can be analyzed by suitably specifying the spatial distribution of the parameters and the details of the relevant spatial coupling mechanisms.

```
% L. Mari, R. Casagrandi, E. Bertuzzo, A. Rinaldo, M. Gatto
% Conditions for transient epidemics of waterborne
% disease in spatially explicit systems
% Royal Society Open Science 6:181517, 2019
% http://dx.doi.org/10.1098/rsos.181517
%
% This MATLAB script can be used to evaluate the asymptotic stability of the
% disease-free equilibrium (DFE) of model (1) described in the main text, as
% well as its generalized reactivity properties associated with the output
% transformation defined in equation (3). It also performs efficient numerical
% simulations of model (1). The script can thus be used to determine conditions
% for endemic/epidemic waterborne disease transmission and to analyze the
% ensuing epidemiological dynamics in fully spatially explicit setting.

clearvars
close all
clc

%%%%%%%%%%%%%%%%%%%%%%%%%%%%%%%%%%%%%%%%%%%%%%%%%%%%%%%%%%%%%%%%%%%%%%%%%%%%%%%%%
% baseline parameter values
% note: see Table 1 in the main text
%%%%%%%%%%%%%%%%%%%%%%%%%%%%%%%%%%%%%%%%%%%%%%%%%%%%%%%%%%%%%%%%%%%%%%%%%%%%%%%%%

N = 1;                                   % population size without disease
mu = 1 / 65 / 365;                       % human mortality rate (1/day)
beta = 1;                                % exposure rate (1/day)
delta = 4e-4;                            % disease-induced mortality (1/day)
gamma = 1 / 5;                           % recovery rate (1/day)
theta = 0.02;                            % rescaled contamination rate (1/day)
```

```
W = 1;                                  % water reservoir size
nu = 1 / 30;                            % pathogen mortality rate (1/day)
l = 1 / 3;                              % pathogen transport rate (1/day)
mS = 0.2;                               % mobility of susceptible human hosts
mI = 0.05;                              % mobility of infected human hosts

%%%%%%%%%%%%%%%%%%%%%%%%%%%%%%%%%%%%%%%%%%%%%%%%%%%%%%%%%%%%%%%%%%%%%%%%%%%%%%%%
% hydrological connectivity and human mobility
% note: P and Q must be defined as (sub-)stochastic matrices of size (n x n)
%%%%%%%%%%%%%%%%%%%%%%%%%%%%%%%%%%%%%%%%%%%%%%%%%%%%%%%%%%%%%%%%%%%%%%%%%%%%%%%%

n = 4;                                  % number of communities
P = [0, 0, 1, 0;                        % hydrological connectivity matrix
     0, 0, 1, 0;
     0.05, 0.05, 0, 0.9;
     0, 0, 0.1, 0];
Q = [0, 0.5, 0.4, 0.1;                  % human mobility matrix
     0.3, 0, 0.5, 0.2;
     1 / 3, 1 / 3, 0, 1 / 3;
     0.2, 0.2, 0.6, 0];

%%%%%%%%%%%%%%%%%%%%%%%%%%%%%%%%%%%%%%%%%%%%%%%%%%%%%%%%%%%%%%%%%%%%%%%%%%%%%%%%
% output transformation
% note: see equation (3) in the main text
%%%%%%%%%%%%%%%%%%%%%%%%%%%%%%%%%%%%%%%%%%%%%%%%%%%%%%%%%%%%%%%%%%%%%%%%%%%%%%%%

cI = 1;                                 % weight of infected hosts
cB = 1;                                 % weight of pathogen concentration

%%%%%%%%%%%%%%%%%%%%%%%%%%%%%%%%%%%%%%%%%%%%%%%%%%%%%%%%%%%%%%%%%%%%%%%%%%%%%%%%
% note: here, all parameters are assumed to be spatially homogeneous, yet this
% script can be used also if some/all parameters are spatially distributed; in
% that case, the community-specific values of each spatially heterogeneous
% parameter must be specified as the nonzero elements of a diagonal matrix
%%%%%%%%%%%%%%%%%%%%%%%%%%%%%%%%%%%%%%%%%%%%%%%%%%%%%%%%%%%%%%%%%%%%%%%%%%%%%%%%

U = diag(ones(n, 1));                   % identity matrix of size n
Z = zeros(n);                           % zero matrix of size n
N = N * U; mu = mu * U;                 % -|
beta = beta * U; delta = delta * U;     %  |
gamma = gamma * U; theta = theta * U;   %  | scalar matrices of size n
W = W * U; nu = nu * U; l = l * U;      %  |
mS = mS * U; mI = mI * U;               % -|
C = [Z, cI * U, Z; Z, Z, cB * U];       % output matrix (equation 3)
clear cI cB

%%%%%%%%%%%%%%%%%%%%%%%%%%%%%%%%%%%%%%%%%%%%%%%%%%%%%%%%%%%%%%%%%%%%%%%%%%%%%%%%
% asymptotic stability
% note: see Appendix A
%%%%%%%%%%%%%%%%%%%%%%%%%%%%%%%%%%%%%%%%%%%%%%%%%%%%%%%%%%%%%%%%%%%%%%%%%%%%%%%%

% build Jacobian matrix at DFE
J0_13 = - ((U - mS) * N + mS * N * Q) * beta;
J0 = [- mu, Z, J0_13;
```

```
      Z, - (mu + delta + gamma), - J0_13;
      Z, theta / W * (U - mI + Q' * mI), - nu - (U - W \ P' * W) * l];
clear J0_13

% evaluate dominant eigenvalue of J0
lambda_max_J0 = max(real(eig(J0)));      % dominant eigenvalue of J0
display(lambda_max_J0);                  % DFE is unstable if lambda_max_J0 > 0

%%%%%%%%%%%%%%%%%%%%%%%%%%%%%%%%%%%%%%%%%%%%%%%%%%%%%%%%%%%%%%%%%%%%%%%%%%%%%%%%
% generalized reactivity
% note: see Appendix B
%%%%%%%%%%%%%%%%%%%%%%%%%%%%%%%%%%%%%%%%%%%%%%%%%%%%%%%%%%%%%%%%%%%%%%%%%%%%%%%%

% build Hermitian matrix at DFE
H0_temp = C * J0 * pinv(C);
H0 = (H0_temp + H0_temp') / 2;
clear H0_temp

% evaluate g-reactivity
[V, D] = eig(H0);                        % eigenvectors and eigenvalues of H0
[lambda_max_H0, maxind] = max(diag(D));  % dominant eigenvalue of H0
display(lambda_max_H0);                  % DFE is g-reactive if lambda_max_H0 > 0
opt_pert_t0 = abs(V(:, maxind));         % optimal perturbation at time 0
clear V D maxind

% human-readable output
if lambda_max_J0 > 0
    msg = ['The DFE is asymptotically unstable (lambda_max_J0 > 0), ' ...
          'thus also g-reactive (lambda_max_H0 > 0)'];
elseif lambda_max_J0 < 0
    if lambda_max_H0 > 0
        msg = ['The DFE is asymptotically stable (lambda_max_J0 < 0), ' ...
               'yet g-reactive (lambda_max_H0 > 0)'];
    else
        msg = ['The DFE is asymptotically stable (lambda_max_J0 < 0), ' ...
               'and non-g-reactive (lambda_max_H0 <= 0)'];
    end
else
    if lambda_max_H0 > 0
        msg = ['The DFE is g-reactive (lambda_max_H0 > 0), ' ...
               'but its asymptotic stability cannot be decided (lambda_max_J0 = 1)'];
    else
        msg = ['The DFE is non-g-reactive (lambda_max_H0 <= 0), ' ...
               'but its asymptotic stability cannot be decided (lambda_max_J0 = 1)'];
    end
end
disp(msg);
clear msg

%%%%%%%%%%%%%%%%%%%%%%%%%%%%%%%%%%%%%%%%%%%%%%%%%%%%%%%%%%%%%%%%%%%%%%%%%%%%%%%%
% model simulation
% note: here, the spatial signature of the optimal perturbation at time 0 with
% an assigned total prevalence of disease in the human population is used as
% initial condition for simulating model (1); different choices can be specified
```

```
% with a different definition of x0
%%%%%%%%%%%%%%%%%%%%%%%%%%%%%%%%%%%%%%%%%%%%%%%%%%%%%%%%%%%%%%%%%%%%%%%%%%%%%%%%

% timespan and initial condition
tspan = [0 60];                          % simulation timespan (days)
prev0 = 0.3;                             % initial total prevalence (%)
Ntot = sum(diag(N));                     % total human population size
x0 = [diag(N);                           % initial condition (3 n x 1 vector)
    opt_pert_t0 / sum(opt_pert_t0(1 : n)) * prev0 / 100 * Ntot];

% numerical integration
[t, x] = ode45(@(t, x) odeSIB(t, x, ...
    N , mu, beta, delta, gamma, theta, W, nu, l, mS, mI, P, Q, n, U), ...
    tspan, x0);
S_t_i = x(:, 1 : n);
I_t_i = x(:, n + 1 : 2 * n);
B_t_i = x(:, 2 * n + 1 :  end);
clear x

% plotting
plot(t, sum(I_t_i,2) / Ntot * 100)
xlabel('Time after perturbation (days)')
ylabel('Total prevalence of infection (%)')

%%%%%%%%%%%%%%%%%%%%%%%%%%%%%%%%%%%%%%%%%%%%%%%%%%%%%%%%%%%%%%%%%%%%%%%%%%%%%%%%
% specification of the model
% note: see system of equations (1) in the main text
%%%%%%%%%%%%%%%%%%%%%%%%%%%%%%%%%%%%%%%%%%%%%%%%%%%%%%%%%%%%%%%%%%%%%%%%%%%%%%%%

function dx = odeSIB(~, x, ...
    N , mu, beta, delta, gamma, theta, W, nu, l, mS, mI, P, Q, n, U)

    % state variables (n x 1 vectors)
    S = x(1 : n);                        % susceptible population
    I = x(n + 1 : 2 * n);                % infected population
    B = x(2 * n + 1 :  end);             % pathogen concentration

    % evaluating some useful rates
    recruitment = diag(mu * N);
    FoI = diag((U - mS + mS * Q) * beta * (B ./ (1 + B)));
    removal = mu + delta + gamma;
    hydrotransport = (U - W \ P' * W) * l;
    contamination = theta / W * ( U - mI + Q' * mI);

    % equations of model (1)
    dSdt = recruitment - (mu + FoI) * S;
    dIdt = FoI * S - removal * I;
    dBdt = contamination * I - (nu + hydrotransport) * B;

    % assembling the function output
    dx = [dSdt; dIdt; dBdt];
end
```

References

Codeço, C. T. (2001). Endemic and epidemic dynamics of cholera: The role of the aquatic reservoir. *BMC Infectious Diseases*, 1:1.

Eisenberg, M., Shuai, Z., Tien, J., and van den Driessche, P. (2013). A cholera model in a patchy environment with water and human movement. *Mathematical Biosciences*, 246:105–112.

Gatto, M., Mari, L., Bertuzzo, E., Casagrandi, R., Righetto, L., Rodriguez-Iturbe, I., and Rinaldo, A. (2012). Generalized reproduction numbers and the prediction of patterns in waterborne disease. *Proceedings of the National Academy of Sciences USA*, 48:19703–19708.

Gatto, M., Mari, L., Bertuzzo, E., Casagrandi, R., Righetto, L., Rodriguez-Iturbe, I., and Rinaldo, A. (2013). Spatially explicit conditions for waterborne pathogen invasion. *The American Naturalist*, 182:328–346.

Horn, R. A. and Johnson, C. R. (1990). *Matrix Analysis.* Cambridge University Press, Cambridge, US.

Kuznetsov, Y. A. (1995). *Elements of Applied Bifurcation Theory.* Springer-Verlag, New York, USA.

Mari, L., Casagrandi, R., Bertuzzo, E., Rinaldo, A., and Gatto, M. (2014). Floquet theory for seasonal environmental forcing of spatially-explicit waterborne epidemics. *Theoretical Ecology*, 7:351–365.

Mari, L., Casagrandi, R., Rinaldo, A., and Gatto, M. (2017). A generalized definition of reactivity for ecological systems and the problem of transient species dynamics. *Methods in Ecology and Evolution*, 8:1574–1584.

Mari, L., Casagrandi, R., Rinaldo, A., and Gatto, M. (2018). Epidemicity thresholds for water-borne and water-related diseases. *Journal of Theoretical Biology*, 447:126–138.

Neubert, M. G. and Caswell, H. (1997). Alternatives to resilience for measuring the responses of ecological systems to perturbations. *Ecology*, 78:653–665.

Silvester, J. R. (2000). Determinants of block matrices. *The Mathematical Gazette*, 84:460–467.

Tien, J. H., Shuai, Z., Eisenberg, M. C., and van den Driessche, P. (2015). Disease invasion on community networks with environmental pathogen movement. *Journal of Mathematical Biology*, 70:1065–1092.

D Supplementary figures

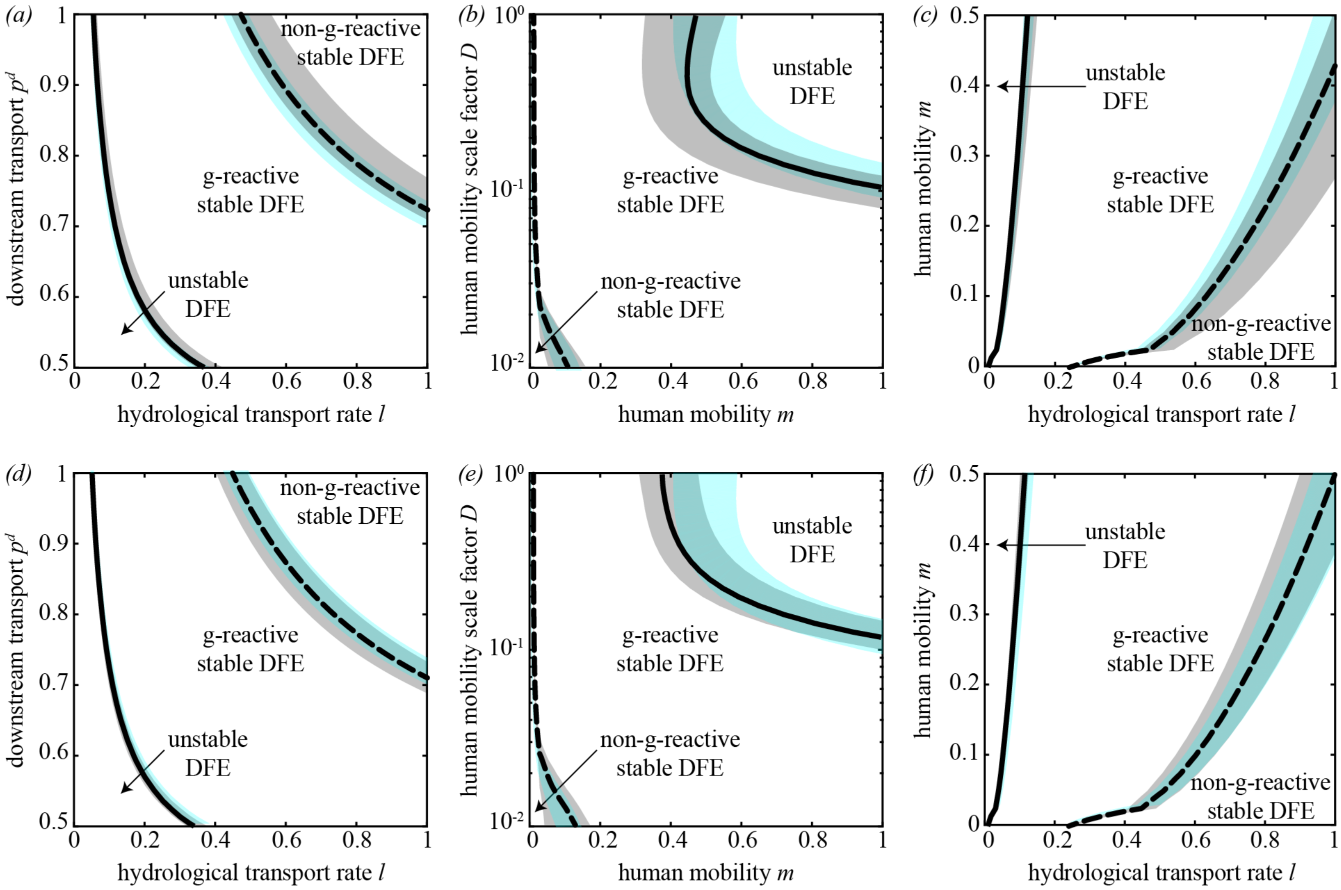

Figure S1: The role of spatial coupling mechanisms in disease epidemicity and endemicity. Details as in Fig. 3 in the main text, for different OCN topologies. Panels *a*–*c* are obtained with OCN configurations like those shown in Fig. 1*a* in the main text, while panels *d*–*f* are obtained with OCN configurations like those shown in Fig. 1*c* in the main text. In all panels, the cyan-shaded areas are the envelopes of the stability/g-reactivity boundaries obtained with OCN configurations like those shown in Fig. 1*b* in the main text, and are reported here for reference.

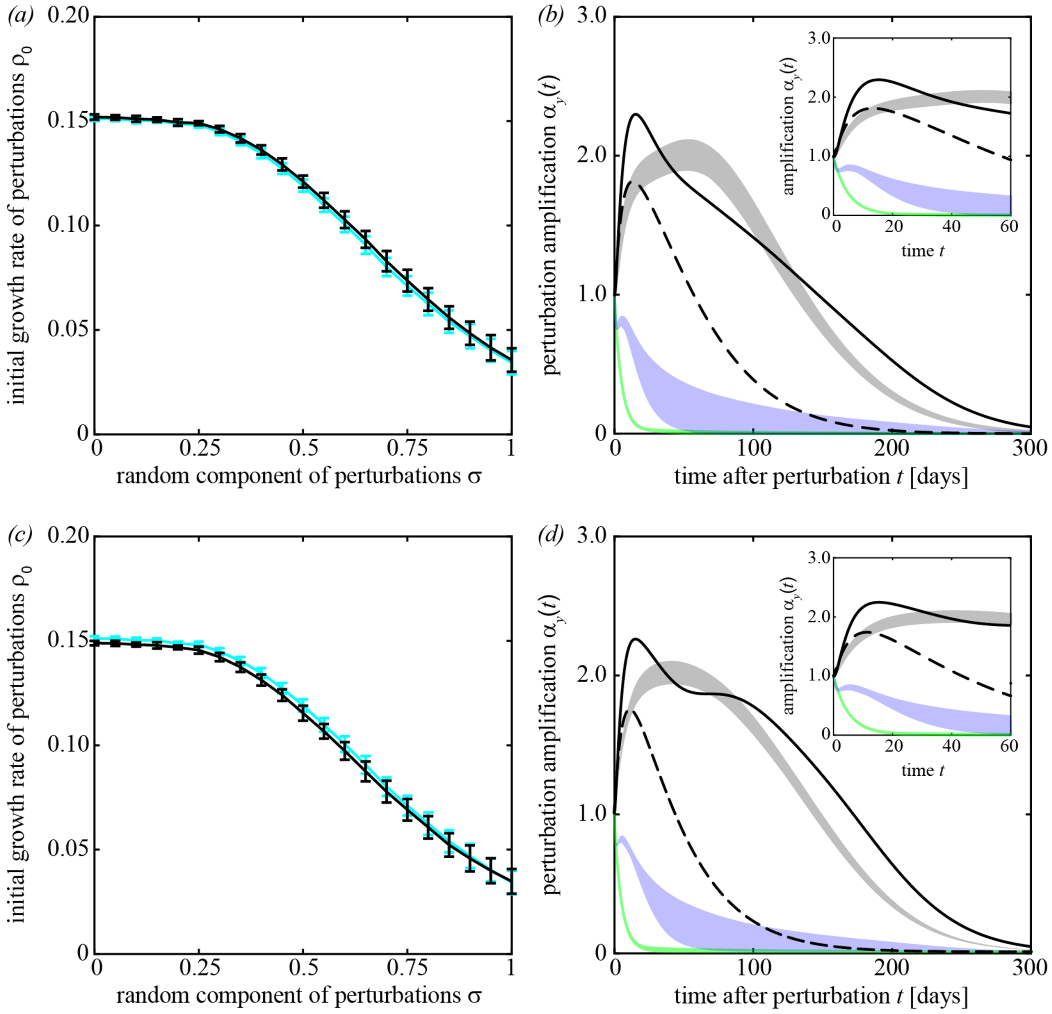

Figure S2: Analysis of short- and long-term amplification of small perturbations to the DFE. Details as in Fig. 4 in the main text, for different OCN topologies. Panels *a*–*b* are obtained with OCN configurations like those shown in Fig. 1*a* in the main text, while panels *c*–*d* are obtained with OCN configurations like those shown in Fig. 1*c* in the main text. The cyan curves in panels *a* and *c* refer to OCN configurations like those shown in Fig. 1*b* in the main text, and are reported here for reference. Panels *c* and *d* are obtained with the leftmost configurations shown in Fig. 1*a* and Fig. 1*c*, respectively.

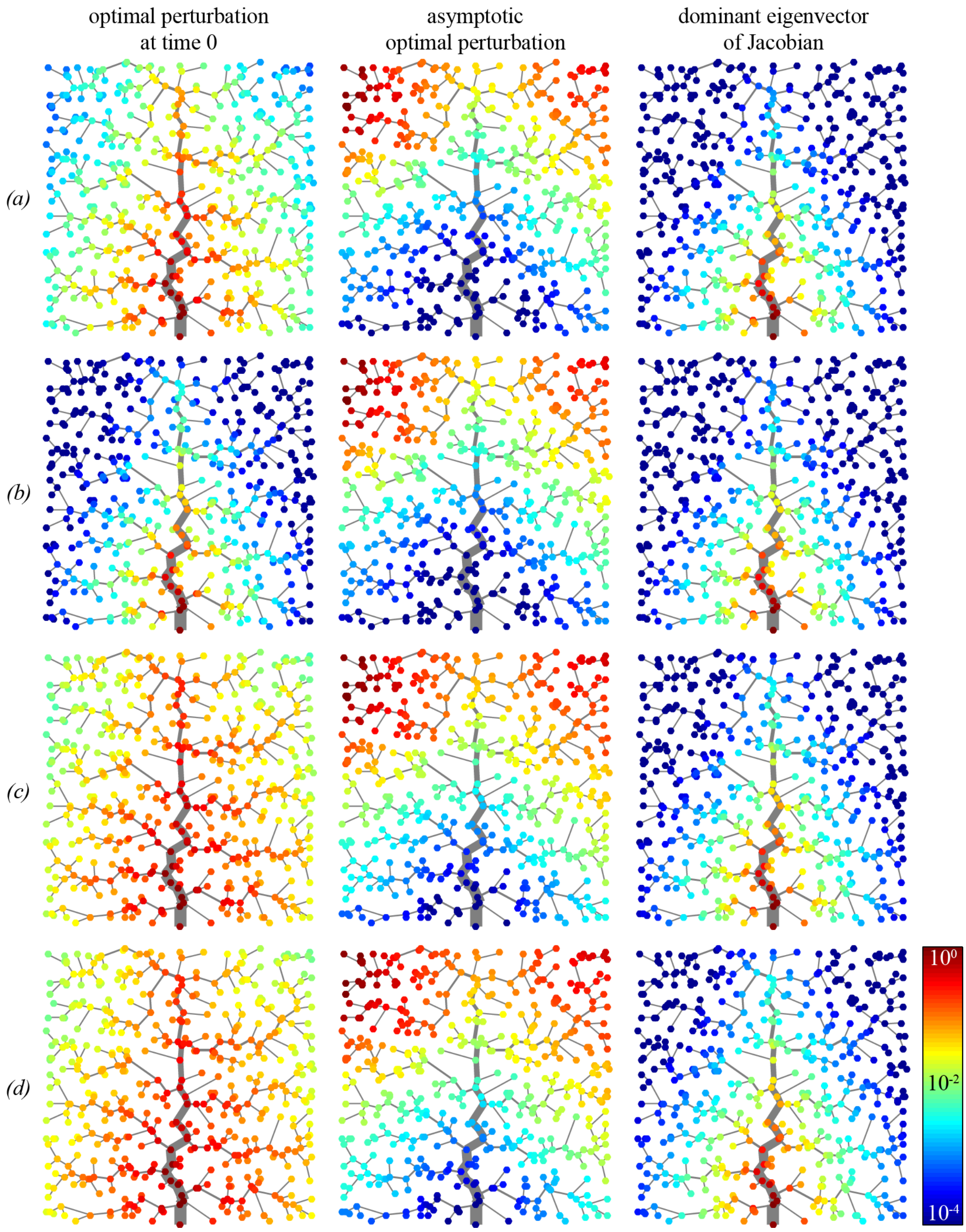

Figure S3: Geography of the optimal perturbations and of disease spread. Details as in Fig. 5 in the main text, for a different OCN topology (the leftmost configuration shown in Fig. 1a) in the main text.

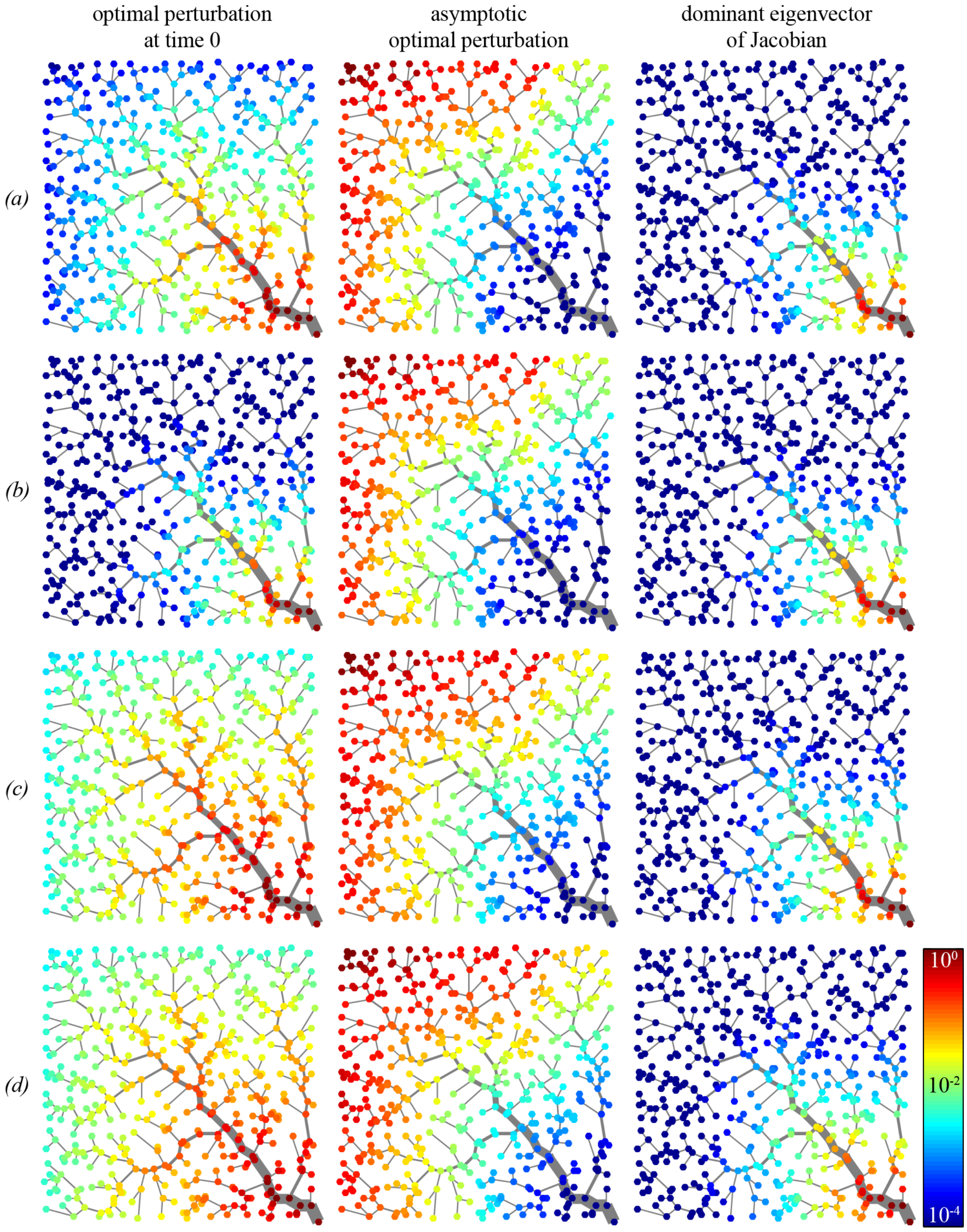

Figure S4: Geography of the optimal perturbations and of disease spread. Details as in Fig. 5 in the main text, for a different OCN topology (the leftmost configuration shown in Fig. 1*c*) in the main text.
